# A Rare Cause of Low Back Pain: Report of a Tailgut Cyst

**DOI:** 10.1155/2012/623142

**Published:** 2012-02-08

**Authors:** E. A. Joyce, D. O. Kavanagh, D. C. Winter

**Affiliations:** Institute for Clinical Outcomes Research and Education (iCORE), St. Vincent's University Hospital, Elm Park, Dublin 4, Ireland

## Abstract

Tailgut cysts, also known as retrorectal cystic hamartomas, are rare developmental abnormalities that typically occur in the retrorectal space. They are believed to arise from remnants of the embryonic hindgut (Hjermstad and Helwig, 1988). They can present as incidental findings during routine examination but over half of patients are thought to present with symptoms. MRI has become the modality of choice to image these frequently misdiagnosed cysts. Biopsy is not recommended. Complete intact surgical excision is advised to avoid the potential complications of these cysts which include infection, fistula formation, and the possibility of malignant transformation (Hjermstad and Helwig (1988), Mathis et al. (2010)). We describe the case of a 46-year-old female who presented with a 6-month history of low back pain. CT and MRI imaging demonstrated a complex retrorectal lesion with supralevator and infralevator components. This was removed using a combined transperineal and transabdominal approach. Histology confirmed a tailgut cyst.

## 1. Case Report

A 46-year-old female was referred with a 6-month history of low back pain. She also reported a vague swelling over the coccyx area that had become apparent to her whilst sitting. She denied any bowel-related symptoms. A colonoscopy, performed one year prior to this to investigate PR bleeding, was normal to the caecum apart from grade I haemorrhoids. A lumbosacral MRI demonstrated a complex lesion in the presacral area likely to represent a dermoid cyst.

**Figure 1:**
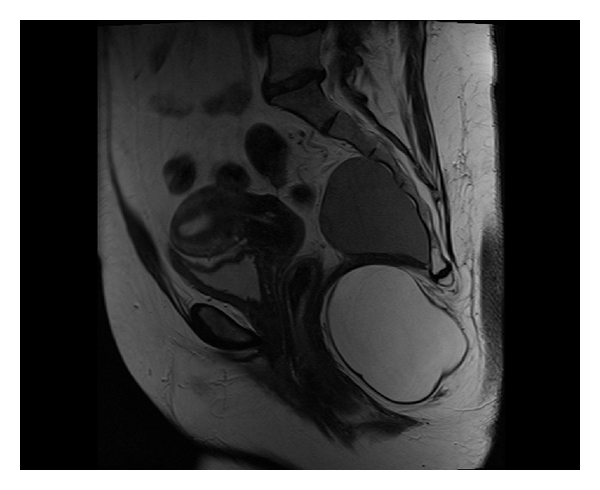
Sagital *T*_2_-weighted MR image demonstrating a bilobed, well-defined cystic lesion in the presacral, precoccygeal and infracoccygeal location.

T2-weighted MR images revealed a bilobed (8.1 cm and 5.8 cm), well defined, cystic lesion, anterior to the sacrococcygeal bones, which bowed the levator ani anteriorly as well as the anal canal and rectum (Figure 1). In between the lobes, there appeared to be a well-defined fibrous band-like structure lying in the plane of the levator ani muscle. CT imaging confirmed no bony involvement. Examination under anaesthesia revealed a soft but well-circumscribed mass in the retrorectal space. The rectal wall was freely mobile over it.

Following discussion of the case at a multidisciplinary team conference, it was decided that surgical excision of this lesion was the most appropriate course of action given its symptomatology and uncertain malignant potential. The procedure was commenced in the lithotomy position. A vertical perineal incision from the tip of the coccyx to the posterior pole of the anal canal/sphincter complex facilitated *en bloc* removal of the infralevator component of the lesion and the coccyx to which it was adherent. This component was not communicating with the supralevator component of the lesion. The latter required a transabdominal approach via a pfannenstiel incision. The peritoneum overlying the pelvic brim was incised posterolaterally, and the mesorectal plane was entered. Dissection was continued in a caudal direction. The rectum was fully mobilised, allowing the cyst, which was adherent to it, to also be mobilised and removed intact. The rectal wall was not breached, and the presacral fascia was fully intact.

Histopathology revealed a multiloculated tailgut cyst containing abundant mucoid material lined by both squamous and glandular mucinous epithelium. The patient had an uneventful postoperative course with complete resolution of her back pain. She remains well at one-year followup with no evidence of recurrence on serial pelvic imaging.

## 2. Discussion

Tailgut cysts, also known as retrorectal cystic hamartomas, are rare congenital lesions that almost invariably occur in the retrorectal space but have been described in prerectal [1] and perirenal [2] locations. The retrorectal space is a potential space bound anteriorly by the mesorectum and posteriorly by the sacrum. The superior border is formed by the peritoneal reflection while the inferior border is formed by the rectosacral fascia. Below the rectosacral fascia lies another potential space, the supralevator space, bound anteriorly by the mesorectum and inferiorly by the levator ani. The lateral borders of the retrorectal space are formed by the ureters, the iliac vessels, the sacral nerve roots, and the lateral stalks of the rectum [3]. The retrorectal space contains loose connective tissue, the middle sacral, iliolumbar and middle haemorrhoidal vessels, branches of the sympathetic and parasympathetic nervous systems, and lymphatics [4].

The true incidence of retrorectal lesions is difficult to establish because there have been few large series published, but it is estimated at 1 in 40,000 based on Mayo Clinic data [5]. The differential diagnoses can be classified as congenital, neurogenic, osseous, miscellaneous, and inflammatory. Excluding inflammatory processes, congenital lesions account for approximately two-thirds of retrorectal lesions [5]. These include developmental cysts, chordomas (remnants of notochord), and anterior sacral meningoceles. Developmental cysts can be further divided according to their origin and histopathological features into tailgut cysts, enteric duplication cysts, dermoid cysts, epidermoid cysts, and teratomas. Middeldorpf described the first report of a retrorectal mass in 1885. He described a rectal duplication cyst in a 1-year-old girl. In the report, he also implied that tumours in this area may be of tailgut origin. *Middeldorpf tumours* became a term applied to any type of congenital cystic presacral tumour, most commonly teratomas, by subsequent early authors. This term is now rarely used because it lacks specificity encompassing a range of cystic tumours [6, 7].

Embryologically, tailgut cysts are believed to arise from vestigial remnants of the embryonic hindgut. The embryo possesses a true tail during early human development which is maximal at the 8 mm stage (35 days gestational age). The primitive hindgut extends into this tail which is caudal to the site of subsequent formation of the anus. Thus, this extension has been termed the postanal gut or tailgut and usually completely regresses by the 35 mm stage of development (56 days gestational age). It is hypothesised that remnants of the tailgut that fail to regress may lead to the subsequent formation of tailgut cysts [6, 8]. The largest reported case series of 53 tailgut cysts over a 35-year period from 1950 to 1985 was described by Hjermstad and Helwig [6]. They found that these cysts predominantly occurred in women (female to male ratio, 3 : 1). The ages ranged from 4 days to 73 years with an average age of presentation at 35 years. There was no correlation between patient age and the size of the lesion at presentation. Half of the patients presented with symptoms. Major presenting features included low back or rectal pain, pain during defecation, rectal fullness, painless rectal bleeding, a change in calibre of stool, and urinary frequency. The average duration of symptoms at presentation was 7.5 months. Our patient had nonspecific lower back pain for 6 months. In the asymptomatic patient group, most lesions were detected incidentally at routine physical or pelvic examination. Most lesions were multicystic and the average diameter was 3.9 cms. They were lined by a variety of epithelia which varied not only among multiple cysts of multicystic lesions but also within the same cyst. The content varied from clear fluid to dense mucous. One patient had a poorly differentiated mucinous adenocarcinoma associated with the tailgut cyst highlighting the malignant potential of these cysts.

Due to the location of tailgut cysts, almost all of them are palpable on rectal examination as extrinsic, contained, fluctuant masses [5, 6]. There are many reports in the literature of these cysts being associated with a postanal midline dimple [6, 9]. This is thought to be due to the traction of the filum terminale on the skin during growth and development. No specific risk factors for the development of tailgut cysts have been documented in the literature. A large proportion of cases are initially misdiagnosed. The diagnosis is often delayed, partially due to the unfamiliarity with this entity but also because the symptoms associated with it often mimic other more commonly occurring pathologies at this site [10]. One should always consider the diagnosis of a tailgut cyst in a middle-aged woman with a history of multiple procedures for recurrences of an anal fistula or abscess.

Plain films, for investigation of presacral masses, are of limited use but may show evidence of bony destruction suggesting malignancy or an osseous lesion. Rarely, they may identify a sacrococcygeal anomaly associated with tailgut cysts. If the patient presents with symptoms such as rectal bleeding, colonoscopy may be helpful to rule out other causes of this. Barium enema rarely contributes additional information apart from characterising the lesion as extrinsic to the rectum [5–7, 11]. Transrectal ultrasound may be useful in demonstrating the integrity of the layers of the rectum as well as revealing a cystic lesion and clarifying whether it is unilocular or multilocular. Occasionally it shows internal echoes due to mucoid material or inflammatory debris [8, 12]. The appearance of a tailgut cyst on CT imaging is usually of a well-defined, thin-walled, uni- or multilocular, nonenhancing lesion in the retrorectal space. Calcification does not tend to be a feature of tailgut cysts but has been reported and if present may suggest the possibility of malignancy [6, 12]. As fistula formation can be a complication of tailgut cysts, a CT fistulogram can demonstrate the contrast filling the cyst but not communicating with a second external opening [13]. MRI has become the modality of choice to image tailgut cysts because of its multiplanar imaging capability (allowing imaging of surgically relevant planes) as well as its good soft tissue contrast. MR imaging typically demonstrates a retrorectal lesion with low signal intensity on T1-weighted images and high signal intensity on-T2 weighted images although this may vary depending on cyst content. Malignancy is suspected if there is focal irregular wall thickening and intermediate signal intensity before contrast on both T1- and T2-weighted images with enhancement after contrast [8, 12, 14, 15].

Preoperative biopsy is indicated if there is a suspicion of malignancy but can be avoided if cystic or clearly benign. The risk of infection of cystic lesions is significantly higher following biopsy especially if performed via a transrectal approach. There is an inherent risk of tract seeding and when sampling transviscerally this has the potential for seeding in another organ. Cyst wall and content biopsies are subject to sampling error [16, 17]. As a result the optimal approach for sampling is a posterior paravertebral approach. If the biopsy is malignant the sampling tract should be excised enbloc when removing the tumor. If this were performed following transrectal sampling it would require excision of a portion of the rectal wall and potentially an abdominoperineal excision. A tumor of uncertain malignant potential should be biopsied as certain pathological subtypes may be optimally treated with neoadjuvant therapy prior to surgery to achieve optimal outcomes. Complete surgical excision is recommended to avoid recurrence, alleviate symptoms, and prevent infection and potential local dissemination of malignant cells. The incidence of malignant degeneration of tailgut cysts in the largest case series was 2% but more recent smaller case series have reported much higher rates [10, 16, 18]. Fistula formation, most commonly to the rectum, has also been reported [16]. Tailgut cysts may become problematic during childbirth causing obstruction of the birth canal and the need to convert to Caesarian section. In fact, this can be a presenting feature of tailgut cysts [19].

Different types of surgical approaches have been described in the literature for the excision of tailgut cysts. These include a posterior approach, an abdominal approach or a combination of the two as described in the current case. The choice of operative approach is determined based on the size of the lesion, its rostral and caudal extent, whether or not it is infected or adherent to the sacrum, pelvic sidewall or adjacent viscera and whether or not malignancy is suspected [3, 16]. The posterior approach is recommended for small benign lesions below the level of the S3 vertebrae [3]. If the superior border of the lesion can be palpated during digital rectal examination, then the posterior approach is likely to be successful [13]. Regardless of the approach, preoperative bowel preparation is required should the need arise to resect part of the rectum if the cyst connects with it. The patient should also be consented, counselled, and marked for a stoma. Blood should be readily available intraoperatively should the need to transfuse arise as haemorrhage from the pelvic vasculature can be difficult to control and potentially catastrophic.

The posterior approach is comprised of many techniques including a transsacrococcygeal, transsacral, transperineal, transsphincteric, and transrectal approaches each with their own advantages depending on the location of the tumour and the surgeon's expertise [3]. The patient can be placed in a jack knife, lithotomy, or lateral position. A parasacrococcygeal, curvilinear, or horizontal incision is used to gain access to the retrorectal space. Division of the anococcygeal ligament facilitates exposure of the tumour. Partial division of the gluteus maximus muscle may also aid this. Routine coccygectomy was previously thought to improve surgical exposure and decrease the risk of recurrence of the cyst but is now only carried out if necessary for complete excision of the cyst or if a malignant lesion is directly invading the coccyx [16, 18]. The cyst should be dissected away from the mesorectum or rectal wall and its lateral attachments. A finger in the rectum provides tactile feedback if the lesion is closely adherent to it to avoid iatrogenic injury. If the cyst cannot safely be removed, then a portion of the rectal wall may need to be excised with it. Every attempt should be made to preserve the sacral nerve roots but if it is necessary to resect them then unilateral preservation of S2–S4 should maintain normal bladder and bowel function [19]. In order to avoid recurrence of infected cysts which have previously been incised and drained, it is important to excise the entire drainage tract and old surgical scar with the specimen [11].

Cysts that have their lowest extent above the level of the S4 vertebrae should be approached transabdominally [3]. Recent case reports have demonstrated laparoscopic excision of these lesions to be a safe and effective approach [20]. The abdominal approach allows direct visualisation of the middle sacral artery, the presacral veins, and the presacral nerves as well as the rectum and ureters [21]. It is usually performed via a lower midline incision followed by mobilisation of the rectum allowing safe dissection of the cyst from other adjacent tissues. Tailgut cysts greater than 4-5 cm are best approached through a combined or abdominosacral approach [19]. This is also true for lesions that extend above and below the level of the S3 vertebrae [3]. A combined approach permits good exposure for protection and dissection of surrounding structures, optimizes vascular control, and allows good visualisation of the cephalad extent of the cyst which may be difficult to judge by the posterior route alone. The blood supply typically arises from the middle sacral artery which can be ligated under direct visualisation prior to removal of the lesion, but larger lesions may require transabdominal control of the iliac vessels [11]. The transrectal approach is not routine for the removal of tailgut cysts and should only be considered in cases of small ruptured transrectal cysts. An intersphincteric approach for small low tumours was described in one small series [21]. This option may avoid the possibility of sacral nerve injury but risks damage to the sphincters [22].

There is no standard recommendation for the followup of tailgut cysts in the literature. Follow-up of this rare condition should therefore be clinical and case specific. If the patient develops symptomatology, targeted cross-sectional imaging should be instituted. In the presence of abnormal histology, serial perineal examination and cross-sectional imaging are advised.

## 3. Conclusion

Complete surgical excision is the treatment of choice for tailgut cysts as this provides a definitive diagnosis, relieves symptoms, and prevents possible complications such as infection, fistula formation, and malignant degeneration [8]. Preoperative imaging with MRI is essential to plan the most appropriate surgical approach. Almost all tailgut cysts can be successfully removed via a posterior approach [5]; however, for larger lesions or suspected malignancy, a combined approach will allow better exposure of the lesion and surrounding structures while allowing optimal vascular control in anticipation of pelvic haemorrhage.
